# Comparison between the Viral Illness Caused by SARS-CoV-2, Influenza Virus, Respiratory Syncytial Virus and Other Respiratory Viruses in Pediatrics

**DOI:** 10.3390/v16020199

**Published:** 2024-01-27

**Authors:** Giulia Brigadoi, Giulia Camilla Demarin, Riccardo Boracchini, Luca Pierantoni, Sara Rossin, Elisa Barbieri, Francesca Tirelli, Anna Cantarutti, Gaia Tempo, Carlo Giaquinto, Marcello Lanari, Liviana Da Dalt, Daniele Donà

**Affiliations:** 1 Division of Pediatric Infectious Diseases, Department for Women’s and Children’s Health, University of Padua, 35128 Padua, Italy; giuliacamilla.demarin@studenti.unipd.it (G.C.D.); carlo.giaquinto@unipd.it (C.G.); daniele.dona@unipd.it (D.D.); 2 Laboratory of Healthcare Research & Pharmacoepidemiology, Unit of Biostatistics, Epidemiology and Public Health, Department of Statistics and Quantitative Methods, University of Milano-Bicocca, 20126 Milan, Italy; riccardo.boracchini@unimib.it (R.B.); anna.cantarutti@unimib.it (A.C.); 3 Pediatric Emergency Unit, IRCCS Azienda Ospedaliero-Universitaria di Bologna, 40138 Bologna, Italy; luca.pierantoni@aosp.bo.it (L.P.); marcello.lanari@unibo.it (M.L.); 4 Pediatric Emergency Department, Department of Women’s and Children’s Health, University of Padua, 35128 Padua, Italy; sara.rossin1988@gmail.com (S.R.); fra.tirelli@gmail.com (F.T.); liviana.dadalt@unipd.it (L.D.D.); 5 Department of Anesthesia and Intensive Care, Fondazione Policlinico Universitario “A Gemelli” IRCCS, 00168 Rome, Italy; gaia.tempo9@gmail.com

**Keywords:** SARS-CoV-2, COVID-19, pediatric, children, respiratory tract infections, respiratory syncytial virus, Influenza virus

## Abstract

Respiratory tract infections (RTIs) are the most common infectious syndromes, primarily caused by viruses. The primary objective was to compare the illness courses between historical RTIs and recent SARS-CoV-2 infections. The study cohort consisted of RTI cases evaluated at the Pediatric Emergency Departments of Padua and Bologna, discharged or admitted with microbiologically confirmed viral RTI between 1 November 2018 and 30 April 2019 (historical period) and 1 March 2020 and 30 April 2021 (recent period). We evaluated the risk of oxygen or respiratory support, hospitalization, antibiotic therapy, and complications among different viral infections. The odds ratio (OR) and the 95% confidence intervals (CIs) were estimated through mixed-effect logistic regression models, including a random intercept on the individual and hospital. We identified 767 RTIs: 359 in the historical period compared with 408 SARS-CoV-2 infections. Infections of SARS-CoV-2 had a lower risk of being admitted (OR 0.04, 95% CI 0.03–0.07), receiving respiratory support (OR 0.19, 95% CI 0.06–0.58), needing antibiotic therapy (OR 0.35, 95% CI 0.22–0.56) and developing complications (OR 0.27, 95% CI 0.14–0.51) compared to all other viral RTIs. COVID-19 in children is clinically similar to other viral RTIs but is associated with a less severe infection course. Thus, most prevention strategies implemented for SARS-CoV-2 should still be considered during RSV and Influenza epidemics.

## 1. Introduction

Respiratory tract infections (RTIs) are the most common infections in the pediatric population and are one of the leading causes of morbidity and mortality in young children worldwide, particularly in low- and middle-income countries [1]. Viral pathogens represent the principal cause of respiratory tract infections, and different viruses usually exhibit different seasonal spreading patterns. The type of virus, host age and pathogen virulence influence the characteristics of different RTIs [2]. Viral respiratory tract infections (VRTIs) decrease with age, with very young children more susceptible [3]. The clinical manifestations of different respiratory viral infections are often similar and may vary from mild illnesses to potentially life-threatening conditions [4]. The most common respiratory pathogens are respiratory syncytial virus (RSV) and Rhinovirus [5], as well as Influenza, Parainfluenza viruses, Adenoviruses, human Metapneumovirus, human Bocavirus and coronaviruses.

Till 2019, RSV and Influenza represented the two viruses with the most significant burden in pediatrics. RSV is the leading cause of respiratory infections in infants [6]. It is the second cause of death globally after malaria in children younger than one year and the first cause of death among respiratory infections. It is responsible for approximately 3 million hospitalizations and 120,000 deaths annually among children under the age of 5 years [7].

Since 2015, Influenza has been responsible for 10,000 hospitalizations in children younger than fourteen years old in Europe and almost 2000 admissions to intensive care units [8]. During the 2019–2020 flu season in Europe, more than 1000 children aged 0–4 years and 800 children aged 5–14 years were admitted to hospital due to Influenza virus infection [9]. Influenza can lead to severe complications, such as pneumonia, bacteremia, and encephalitis [9]. It can spread rapidly in school settings, leading to outbreaks and increased absenteeism. School closures during severe flu seasons can significantly impact children’s education and parents’ ability to work [9].

At the end of 2019, a novel coronavirus emerged called Severe Acute Respiratory Syndrome Coronavirus 2 (SARS-CoV-2), causing coronavirus disease 2019 (COVID-19).

In the European pediatric population, according to the last update (week 47, 2023) reported by the European Center for Disease Prevention and Control, there were nearly 26 million COVID-19 cases in children [10].

Many strategies were employed to mitigate the transmission of the virus. These strategies encompassed preventive measures such as maintaining physical distance between individuals, mandating face coverings, and emphasizing regular hand hygiene. These measures effectively reduced the spread of the SARS-CoV-2 virus and consequently impacted the prevalence of other viral infections, decreasing the burden of acute respiratory illnesses [11,12,13,14]

This study aimed to explore the associations between viral respiratory pathogens and illness course in the pediatric population in Italy, comparing SARS-CoV-2 infection with other respiratory viruses in terms of hospitalization, length of hospital stay, need for respiratory support, use of antibiotics or clinical complications.

## 2. Materials and Methods

### 2.1. Study Design and Population

This multi-center observational retrospective study was conducted at the Department of Women’s and Children’s Health of the University of Padua and the Pediatric Emergency Unit of the IRCCS Azienda Ospedaliero-Universitaria of Bologna, from 1 November 2018 to 30 April 2021.

The study cohort consisted of all viral respiratory tract infections (RTIs) of children aged 0 to 15 years discharged from the Pediatric Emergency Department (PED) or admitted to the Pediatric Acute Care Unit (PACU) with microbiologically confirmed viral RTIs (International Classification of Diseases, 9th Revision, Clinical Modification code or descriptive) for Influenza virus, RSV, Adenovirus, Rhinovirus, Metapneumovirus, other coronaviruses, and SARS-CoV-2 infections. Polymerase chain reaction (PCR) analysis of nasal and oropharyngeal swabs was performed according to the internal protocol of Padua and Bologna hospitals for the detection of the different viruses.

Cases without a laboratory-confirmed infection, with two or more detected viruses or with concurrent bacterial infection were excluded from the analysis.

To avoid possible misclassification, the collection of RTIs caused by viruses other than SARS-CoV-2 was performed in a historical period between 1 November 2018 and 30 April 2019; SARS-CoV-2 infections were captured from 1 March 2020 to 30 April 2021 (recent period).

Included cases were further categorized into two groups: outpatients (discharged home after PED visit) or inpatients (hospitalized at the PACU).

For healthcare resource analysis, we independently evaluated the respiratory supports provided in outpatient and inpatient settings.

### 2.2. Definition of Exposure

RTIF viruses of interest were classified based on the result of the swab performed on arrival at the emergency room.

Comparisons of interest were (A) SARS-CoV-2 RTIs vs. others, (B) SARS-CoV-2 RTIs vs. Influenza RTIs, (C) SARS-CoV-2 RTIs vs. RSV RTIs, (D) SARS-CoV-2 RTIs vs. Adenovirus RTIs and (E) SARS-CoV-2 RTIs vs. Rhinovirus RTIs, Metapneumovirus RTIs and other coronavirus RTIs.

### 2.3. Definition of the Outcomes of Interest

To explore the association between viruses and illness course, the following outcomes were considered: the number of cases (i) requiring oxygen therapy, especially low-flow oxygen (nasocannula) and high-flow oxygen or mechanical ventilation (both non-invasive and invasive); (ii) requiring antibiotic therapy; (iii) requiring hospitalization for more than 3 days; and (iv) experiencing complications (for example, neutropenia, pancytopenia, etc.).

Oxygen therapy was initiated when oxygen saturation fell below 92–94% on room air. The decision of which ventilatory support to administer (low-flow oxygen, high-flow oxygen, non-invasive or invasive mechanical ventilation) was left to the physician, based on the patient’s clinical condition.

### 2.4. Data Source and Collection

Corporate information systems were used to obtain all the cases evaluated at the PEDs during the specific time periods mentioned above. Medicine use and clinical and demographic data for all patients were extracted from electronic medical records and manually entered into the database using REDCap^®^ (version 11.1.2—2024 Vanderbilt University) data collection forms intentionally designed for the condition.

A study survey number was assigned to each patient to ensure data privacy. No personally identifying data were collected.

### 2.5. Statistical Analysis

Baseline socio-demographic and clinical characteristics were summarized descriptively through medians and interquartile range (IQR) for continuous variables and frequency distributions for categorical variables overall and among viral RTIs. The Chi-square, exact Fisher and Wilcoxon tests were used as appropriate. All descriptive analyses were conducted on the overall population separately for each hospital (i.e., Padua or Bologna) and outpatient or inpatient setting. Multivariate logistic regression models, adjusted by age, gender, comorbidities and ethnicity, were used to estimate the odds ratio (OR) and the corresponding 95% confidence interval (95% CI) for each contrast of interest. We included a random intercept in the hospital to take into account the possible differences in management.

## 3. Results

### 3.1. Study Population

The study cohort included 767 viral RTIs (377 in Padua and 390 in Bologna): 359 in the historical period compared with 408 SARS-CoV-2 infections.

The socio-demographic and clinical characteristics of the cohort are shown in Table 1 and Table S1, stratified by center. Considering the overall cases, the median age at infection was 15.77 months, with younger cases of RTIs in Padua than in Bologna (12.25 months and 19.77 months, respectively, *p* = 0.0006)

However, this difference persisted only in the outpatient cases (22.95 months in Padua and 75.67 months in Bologna, *p* < 0.0001), while the median age of the inpatient cases was similar (5.83 months in Padua and 6.83 months in Bologna, *p* = 0.31). SARS-CoV-2 cases were usually older compared to the other RTI cases (46.46 months, [7.93–120.98]), while RSV cases were usually younger (3.25 months [1.52–9.33]) (Table 1).

Overall, 71.19% of cases had no comorbidities, with no differences in the two centers (72.68% in Padua and 69.74% in Bologna, *p* = 0.37). Prematurity, defined as birth before 37 weeks of gestation, accounted for 10.17% of all cases, with no significant differences in the overall rate in the two centers (8.22% in Padua and 12.05% in Bologna, *p* = 0.08) (Table 1). However, most of these cases were hospitalized at the Padua center (26/31), while many cases of them were discharged home from the Bologna center (18/47) (Supplementary Table S1).

Considering overall infections, SARS-CoV-2 and RSV were the most common viruses isolated in both centers, with more RSV cases in the Bologna center than the Padova center (Supplementary Table S2). Almost all cases with RSV infection were hospitalized, except for two cases in the Padua Center. However, when considering the SARS-CoV-2 infections, most cases were treated as outpatients, especially in the Bologna center (Supplementary Table S2). Other viruses such as Adenovirus, Rhinovirus and Influenza virus were less common, with some variations in the number of cases in the two centers. Rhinovirus, in particular, was more frequently isolated in the Padua center than in the Bologna centers.

### 3.2. Patient Signs and Symptoms

Considering overall infections, fever, rhinitis, cough and poor feeding were the most common symptoms (61.54%, 45.24%, 50.20% and 30.38%, respectively). Gastrointestinal symptoms were also reported, especially nausea or vomiting, diarrhea and abdominal pain, but were less common (Table 2).

Fever was reported in almost more than half of cases caused by different viruses; instead, other signs and symptoms reported had some differences between the various viral infections (Table 2).

Among SARS-CoV-2-positive cases, 57.84% experienced fever, while rhinitis and cough were less frequent than in other infections (29.9% and 27.21%, respectively) and dyspnea was rarely reported (4.17%). In contrast, RSV cases experienced less frequent fever (51.96%), but more commonly presented with rhinitis, cough, dyspnea and poor feeding (72.09%, 86.27%, 50.49% and 65.20%, respectively) (Table 2).

Other respiratory viruses also commonly presented fever, rhinitis and cough, followed by poor feeding, dyspnea and gastrointestinal signs and symptoms in some cases.

The systolic and diastolic blood pressure, heart and respiratory rate and oxygen saturation are reported in Tables S3 and S4, stratified by center, virus and type of management setting (outpatient or inpatient). The two centers had no significant differences in the clinical characteristics’ distribution for most of the outcomes of interest. For inpatients, the median days of hospitalization were also reported and were similar between the two centers for all the viruses (4 days in Padua and 5 days in Bologna, [3,4,5,6,7], *p* = 0.52). Considering the specific type of virus, RTI cases caused by RSV and Influenza ha a longer hospitalization course than the other viruses.

### 3.3. Clinical Outcomes

Considering both centers, 110/767 (14.80%) cases required oxygen therapy (nasal cannula or mask), and 64/767 (8.34%) needed high-flow nasal cannula (HFNC). Most of them had RSV infections. Only 13/767 (1.71%) required mechanical ventilation. Clinical outcomes are summarized in Table 3.

There were 204 cases of RSV infections. Of these, 69/204 (36.32%) needed O2 therapy, 55/204 (26.96%) needed high flow and 7/204 (3.43%) required mechanical ventilation.

Considering SARS-CoV-2 RTIs, only 10/408 (2.49%) needed O2 therapy, 4/408 (0.98%) needed high flow and 3/408 (0.74%) required mechanical ventilation.

Overall, 4/44 (9.30%) of all RTIs positive for influenza virus needed O2 therapy, and 1/44 (2.27%) used high flow.

Differences in the use of resources in the two centers are reported in Table S5.

### 3.4. Illness Courses

**Figure 1:**
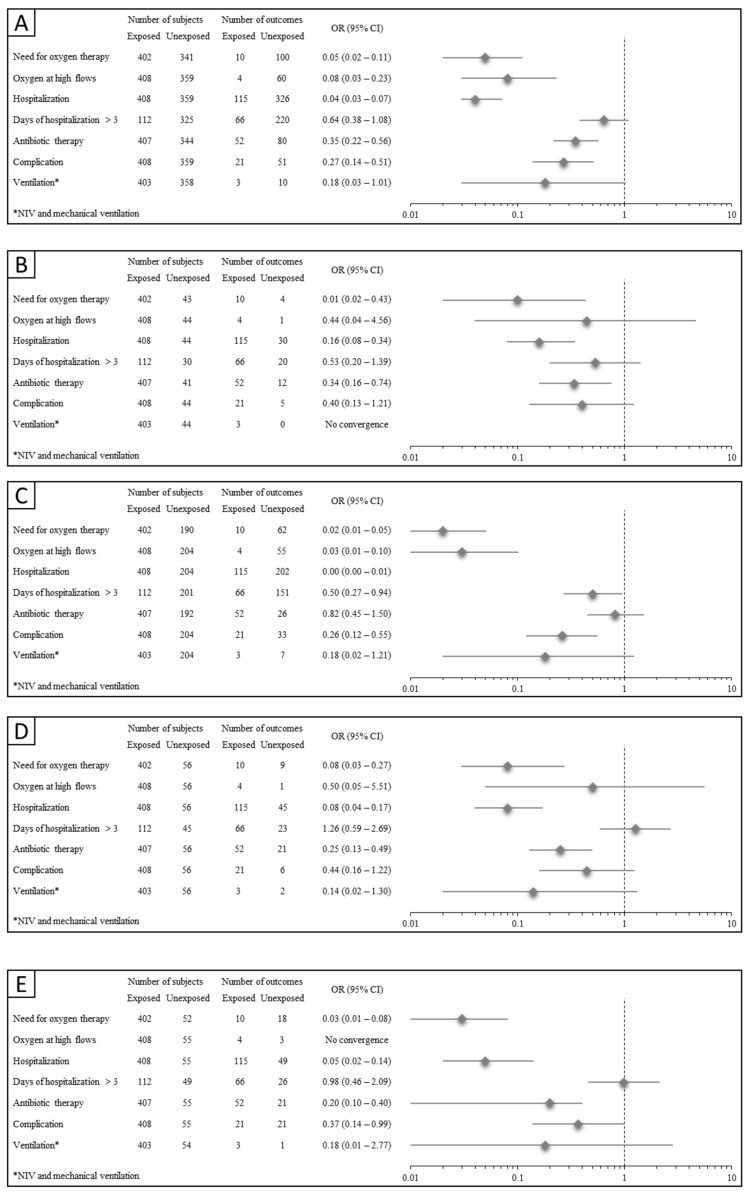
Odds ratios (and 95% confidence intervals) of outcomes of interest associated with SARS-CoV-2 infections. Adjustment by age, sex, comorbidities and ethnicity. (**A**) COVID-19 vs. all other RTIs; (**B**) COVID-19 vs. Influenza; (**C**) COVID-19 vs. RSV; (**D**) COVID-19 vs. Adenovirus; (**E**) COVID-19 vs. Rhinovirus.

The adjusted odds ratios (ORs) for the association between SARS-CoV-2 exposure compared to other overall and specific viral RTIs and all outcomes of interest are reported in Figure 1. SARS-CoV-2 infections had a significantly reduced risk of (i) need for oxygen therapy, ranging from 92% (Panel D) to 99% (Panel B); (ii) oxygen at high flow, ranging from 97% (Panel C) to 92% (Panel A); (iii) hospitalization, ranging from 84% (Panel B) to 96% (Panel A); (iv) being hospitalized for more than three days, 50% (Panel C); (v) using antibiotic therapy, ranging from 65% (Panel A) to 80% (Panel E); and (vi) complications, ranging from 74% (Panel C) to 63% (Panel E).

No significant associations were found in studying the need for invasive ventilation with all the exposure definitions.

## 4. Discussion

This Italian retrospective cohort study, conducted in two pediatric settings, compares the clinical characteristics of SARS-CoV-2 RTIs with those of other respiratory viruses in children and infants, with the aim of improving our understanding of the duration and severity of symptoms in viral respiratory tract infections.

Aside from SARS-CoV-2, the most commonly detected respiratory viruses in children were RSV, followed by Adenovirus, Rhinovirus, Influenza virus, Metapneumovirus and Coronaviruses [5,15].

In line with previous studies [5,15], we observed that younger children were more likely to be infected with respiratory viruses than older children. RSV was more frequently detected in children under one year of age, while SARS-CoV-2, Adenovirus, Influenza virus and Rhinovirus were detected across all age groups.

Respiratory viral infections often present with similar symptoms, and clinical manifestations can range from mild to severe illness. Indeed, as previously stated, most children with COVID-19 experienced fever > 37.5 °C, rhinitis and cough [16,17,18,19], while concomitant gastrointestinal symptoms were less reported [20]. Moreover, we found no significant differences in clinical manifestations among viruses, which is consistent with previous research. Liu et al. (2020) [21] discovered clinical similarities between children infected with SARS-CoV-2 and Influenza A or B viruses. Similarly, Esposito et al. (2013) [22] examined 17 different respiratory viruses and did not find differences in clinical manifestations.

Our data revealed that younger children with RSV infections were hospitalized more frequently than older children. Furthermore, although not requiring additional oxygen or ventilatory support, the median age of children admitted to the PACU with SARS-CoV-2 infection was lower than that of those released. Indeed, when comparing the clinical data of patients infected with SARS-CoV-2 RTIs to those with other viruses, children with COVID-19 appeared to have a milder clinical presentation: their body temperature upon admission to the hospital was lower, with a median oxygen saturation of 99% (99–100%) in room air, and 96.07% of them did not require oxygen therapy or ventilatory support.

SARS-CoV-2 infection was not associated with a more severe course of illness than other viruses. Our data showed that SARS-CoV-2 infection had a significantly reduced risk of need for oxygen therapy, high-flow nasal cannula and admission to the hospital or to intensive care units. Other viral infections, such as respiratory syncytial virus infection, have a more severe course, with an increased need for ventilatory support (both non-invasive and invasive).

In our study, children with SARS-CoV-2 infections received fewer antibiotics than children with other viral infections. Secondary bacterial infections are rare in patients with SARS-CoV-2, while they are more common in patients with other viral infections, such as Influenza. We did not collect the specific type of antibiotics administered, neither the appropriateness of prescription; however, more effort should be made to reduce antibiotic prescriptions in children with viral infections [23,24].

Active prevention strategies and therapies are only available for some of these viruses. An effective vaccine against Influenza is available every year. However, the rate of vaccination in Italy, both in pediatric and adult populations, is very low (20% of the total population) [25]. An effective antiviral treatment is available for Influenza: oseltamivir can be prescribed in children with Influenza virus infection if detected in the first 48 h of symptoms or if they require hospital admission [26]. Regarding RSV, two different preventive strategies, both monoclonal antibodies, are available for children: palivizumab and nirsevimab. Both strategies seem to be effective in preventing the disease. Palivizumab is only available in Italy for targeted populations, such as preterm infants and infants with comorbidities. Conversely, Nirsevibam has showed efficacy both for preterm and term infants and is recommended for both groups before the start of their first RSV season [27]. Although Nirsevimab has been approved by EMA, it is not yet available in Italy. Routine use of antiviral therapy with Ribavirin is not recommended outside of HSCT and lung transplants [28,29].

Nowadays, there are different vaccination and therapeutic strategies that seem to be effective against SARS-CoV-2 [30,31], but not all these are available for children and almost none of them were available during the first year of the pandemic. The first vaccine was authorized in December 2020, and many other vaccines were licensed in the years after, but for children between 5 and 11 years, only a few vaccines were authorized from the end of 2021 [32]. Different therapeutic strategies, both monoclonal antibodies and antiviral treatments, are available. Nevertheless, specific monoclonal antibodies are effective against specific variants of SARS-CoV-2 and do not work any more against the new variants [31]. Instead, antiviral treatment seems to be still effective against the new variants of SARS-CoV-2, but only remdesivir is approved for pediatric age [33]. There are currently very little data about the efficacy of this treatment in children, and more studies with high-quality evidence are needed.

In light of the above, it becomes evident that the changing epidemiology of common respiratory pathogens is mainly due to stringent non-pharmacological public health interventions, with more attention paid to hygiene, routine use of masking and physical distance [34,35]. Indeed, during the pandemic, measures such as lockdowns and school closures were implemented, and remote work arrangements were facilitated. Since introducing effective vaccines in 2021, the pandemic has been characterized by less restrictive measures and more social interactions. Consequently, the circulation of common infectious pathogens within pediatric communities has increased. Countries like Australia and New Zealand, which quickly reopened their communities to everyday pre-pandemic life, experienced an unexpected seasonal surge of bronchiolitis with many cases compared to pre-pandemic periods [34]. Researchers worldwide are concerned about the potential for new, more severe RSV epidemics due to a so-called “immunity debt”: the reduced circulation of all viral pathogens due to extended periods of low exposure to these agents may provoke an absence of immune experience, lower immune stimulation and a higher susceptibility to future, potentially more severe, viral infections. Other than expected, data suggest that although there were more RSV infections, they were not more severe than in previous years [35]. According to Camporesi et al. [34], the expected 2021–2022 bronchiolitis season in Italy started and peaked earlier than the usual pre-pandemic seasons but had a shorter duration and was not more severe. Therefore, the data show an increased virulence but not aggressivity of RSV after the SARS-CoV-2 pandemic period.

Thus, even after the COVID-19 pandemic has ended [36], nonpharmacological preventive hygiene measures should be proposed and implemented to reduce virus spread, at least in healthcare settings. Recent results suggest that social distancing decreases the spreading of common respiratory viral diseases and reduces the need for PED visits and hospitalization among children [37]. Various preventive strategies have proven effective in mitigating the spread of these respiratory infections and should be strongly considered during outbreaks, including vaccinations, hand hygiene, face masks, physical distancing, environmental cleaning and disinfection.

This study presented some limitations. First, confidence intervals were wide, so further studies are required to understand better the clinical differences between SARS-CoV-2 and other respiratory viruses. Second, it needs to be considered that all SARS-CoV-2-infected patients were enrolled during the first and second waves. During this time, the most common SARS-CoV-2 variants in Italy were Alpha and Gamma. We did not collect data about the Delta and Omicron variants that spread in Italy after May 2021. At the same time, the Delta variant of concern showed a higher tendency to be linked with upper respiratory tract symptoms and the Omicron variant of concern was more likely to be linked to upper respiratory and gastrointestinal symptoms in contrast to earlier variants [38]. Indeed, hospitalization rates seem to be higher in patients with RSV than the SARS-CoV-2 Omicron variant, as demonstrated by a recent study published in December 2023 [39]. New variants of concern may still emerge, potentially affecting their characteristics and their outcomes, especially in pediatric populations.

## 5. Conclusions

Although COVID-19 in infants and children is clinically similar to other viral respiratory tract infections, it seems to be associated with a less severe infectious course. Most hygiene measures and prevention strategies adopted and implemented for SARS-CoV-2 should also be strongly considered during RSV, Influenza and Adenovirus epidemics, especially in pediatric settings, to reduce the morbidity of infants and children.

## Figures and Tables

**Table 1:** Socio-demographic and clinical characteristics stratified by virus.

	Overall	SARS-CoV-2	RSV	Adenovirus	Rhinovirus	Influenza A and B	Metapneumovirus	Coronavirus
	*n* = 767	*n* = 408	*n* = 204	*n* = 56	*n* = 32	*n* = 44	*n* = 17	*n* = 6
**Socio-demographic characteristics**								
*Age*—mo. Median (p25–p75)	15.77 (3.44–69.80)	46.46 (7.93–120.98)	3.25 (1.52–9.33)	22.31 (12.11–39.54)	5.77 (1.23–76.70)	18.59 (4.70–31.05)	10.03 (6.07–24.69)	4.93 (1.90–17.41)
*Gender*								
Female	345 (44.98)	182 (44.61)	95 (46.57)	27 (48.21)	12 (37.50)	21 (47.73)	6 (35.29)	2 (3.33)
Male	422 (55.02)	226 (55.39)	109 (53.43)	29 (51.79)	20 (62.50)	23 (52.27)	11 (64.71)	4 (66.67)
*Ethnicity*								
Caucasian	597 (77.84)	316 (77.45)	151 (74.02)	47 (83.93)	28 (87.50)	39 (88.64)	13 (76.47)	3 (50)
Other	170 (22.16)	92 (22.55)	53 (25.98)	9 (16.07)	4 (12.50)	5 (11.36)	4 (23.53)	3 (50)
Pediatric visit 3 days previous	151 (19.69)	15 (3.68)	75 (36.76)	20 (35.71)	11 (34.38)	17 (38.64)	8 (47.06)	5 (83.33)
Sent by pediatrician	99 (12.91)	26 (6.37)	387 (18.63)	13 (23.21)	4 (12.50)	11 (25)	6 (35.29)	1 (16.67)
*Comorbidities*								
No comorbidities	546 (71.19)	293 (71.81)	145 (71.08)	38 (67.86)	21 (65.63)	32 (72.73)	12 (70.59)	5 (83.33)
At least one comorbidity	221 (28.8)	115 (28.19)	59 (28.9)	18 (32.14)	11 (34.38)	12 (27.27)	5 (29.41)	11 (16.67)
Most frequent comorbidities								
Prematurity	78 (10.17)	32 (7.84)	34 (16.67)	3 (5.36)	5 (15.63)	4 (9.09)	0 (0)	0 (0)
Chronic neurologic disease	22 (2.87)	9 (2.21)	5 (2.45)	4 (7.14)	3 (9.38)	0 (0)	0 (0)	1 (16.67)
Onco-hematological disease	17 (2.22)	11 (2.70)	2 (0.98)	1 (1.79)	1 (3.13)	1 (2.27)	1 (5.88)	0 (0)

**Table 2:** Signs and symptoms at the admission to the PEDs.

	Overall	SARS-CoV-2	RSV	Adenovirus	Rhinovirus	Influenza A and B	Metapneumovirus	Coronavirus
	*n* = 767	*n* = 408	*n* = 204	*n* = 56	*n* = 32	*n* = 44	*n* = 17	*n* = 6
Symptoms								
Fever > 37.5 °C	472 (61.54)	236 (57.84)	106 (51.96)	50 (89.29)	23 (71.88)	39 (88.64)	14 (82.35)	4 (66.67)
Rhinitis	347 (45.24)	122 (29.90)	147 (72.09)	23 (41.07)	20 (62.50)	20 (45.45)	12 (70.59)	3 (50)
Cough	385 (50.20)	111 (27.21)	176 (86.27)	28 (50)	20 (62.50)	31 (70.45)	16 (94.12)	3 (50)
Dyspnea	164 (21.38)	17 (4.17)	103 (50.49)	13 (23.21)	11 (34.38)	7 (15.91)	8 (47.06)	5 (83.33)
Earache	16 (2.09)	10 (2.45)	0 (0)	3 (5.36)	1 (3.13)	1 (2.27)	1 (5.88)	0 (0)
Conjunctivitis	16 (2.09)	9 (2.21)	2 (0.98)	2 (3.57)	1 (3.13)	2 (4.55)	0 (0)	0 (0)
Weakness	19 (2.48)	11 (2.70)	0 (0)	2 (3.57)	1 (3.13)	5 (11.36)	0 (0)	0 (0)
Mental confusion, drowsiness	13 (1.69)	2 (0.49)	3 (1.47)	1 (1.79)	6 (18.75)	1 (2.27)	0 (0)	0 (0)
Abdominal pain	40 (5.22)	29 (7.11)	3 (1.47)	6 (10.71)	1 (3.13)	1 (2.27)	0 (0)	0 (0)
Nausea/vomiting	99 (12.91)	50 (12.25)	23 (11.27)	7 (12.50)	5 (15.63)	7 (15.91)	6 (35.29)	1 (16.67)
Diarrhea	72 (9.39)	49 (12.01)	9 (4.41)	6 (10.71)	4 (12.50)	3 (6.82)	1 (5.88)	0 (0)
Poor feeding	233 (30.38)	32 (7.84)	133 (65.20)	27 (48.21)	12 (37.50)	20 (45.45)	7 (41.18)	2 (33.33)
Lymphadenopathy	33 (4.30)	6 (1.47)	2 (0.98)	11 (19.64)	2 (6.25)	11 (25)	1 (5.88)	0 (0)
Skin rash	28 (3.65)	13 (3.49)	5 (2.45)	4 (7.14)	1 (3.13)	5 (11.36)	0 (0)	0 (0)
Lung crackles	9 (1.17)	3 (0.74)	3 (1.47)	2 (3.57)	0 (0)	1 (2.27)	0 (0)	0 (0)
Other	97 (12.65)	44 (10.78)	20 (9.80)	13 (23.21)	8 (25)	8 (18.18)	2 (11.76)	2 (33.33)

**Table 3:** Clinical outcomes.

	Overall	SARS-CoV-2	RSV	Adenovirus	Rhinovirus	Influenza A and B	Metapneumovirus	Coronavirus
	*n* = 767	*n* = 408	*n* = 204	*n* = 56	*n* = 32	*n* = 44	*n* = 17	*n* = 6
*Outcome*								
Need for O2 therapy	110 (14.80)	10 (2.49)	69 (36.32)	9 (16.07)	10 (32.26)	4 (9.30)	5 (33.33)	3 (50)
O2 high flow	64 (8.34)	4 (0.98)	55 (26.96)	1 (1.79)	2 (6.25)	1 (2.27)	0 (0)	1 (16.67)
Admission	441 (57.50)	115 (28.19)	202 (99.02)	45 (80.36)	28 (87.50)	30 (68.18)	15 (88.24)	6 (100)
Days of hospitalization > 3	286 (65.45)	66 (58.93)	151 (75.12)	23 (51.11)	15 (53.57)	20 (66.67)	10 (66.67)	1 (16.67)
Antibiotic therapy	132 (17.58)	52 (12.78)	26 (13.54)	21 (37.50)	12 (37.50)	12 (29.27)	6 (35.29)	3 (50)
Complication	72 (9.39)	21 (5.15)	33 (16.18)	6 (10.71)	4 (12.50)	5 (11.36)	3 (17.65)	0 (0)
Mechanical ventilation	13 (1.71)	3 (0.74)	7 (3.43)	2 (3.57)	1 (3.23)	0 (0)	0 (0)	0 (0)

## Data Availability

The data used in this study cannot be made publicly available due to Italian data protection laws. The anonymized datasets generated during and/or analyzed during the current study can be provided on reasonable request, from the corresponding author, after written approval by the Ethical committees.

## Supplementary Materials

The following supporting information can be downloaded at: https://www.mdpi.com/article/10.3390/v16020199/s1. Table S1. Socio-demographic and clinical characteristics stratified by centre and setting; Table S2. Virus distribution stratified by center and setting; Table S3. Continuous clinical characteristics’ distribution stratified by centre and setting; Table S4. Clinical characteristics at admission to the PEDs stratified by centre, setting and type of virus; Table S5. Use of resources distribution.
