# Supplementary material for: Comparison between the Viral Illness Caused by SARS-CoV-2, Influenza Virus, Respiratory Syncytial Virus and Other Respiratory Viruses in Pediatrics
Source: Viruses. 2024 Jan 27;16(2):199. doi: 10.3390/v16020199 (PMC10891516; doi:10.3390/v16020199)
Supplement: Supplementary file 1 [file viruses-16-00199-s001.zip › viruses-2842630-supplementary.pdf]

Table S1. Socio-demographic and clinical characteristics stratified by centre and setting

	Overall N (%)		P- value	Inpatients N (%)		P- value	Outpatients N (%)		P- value
	**Padova**	**Bologna**		**Padova**	**Bologna**		**Padova**	**Bologna**	
	**(N=377)**	**(N=390)**		**(N=238)**	**(N=203)**		**(N=139)**	**(N=187)**	
**Socio-demographic characteristics**									
Age – mo. Median (p25 – p75)	12.25 (2.21- 52.57)	19.77 (4.62 – 92.39)	0.0006	5.83 (1.57 – 40.13)	6.83 (2.62 – 24.16)	0.3119	22.95 (7.44 – 86.30)	75.67 (16.46 – 129.18)	< 0.0001
**Gender**									
F	184 (48.81)	161 (41.28)	0.0362	120 (50.42)	82 (40.39)	0.0352	64 (46.04)	79 (42.25)	0.4944
M	193 (51.19)	229 (58.72)		118 (49.58)	121 (59.61)		75 (53.96)	108 (57.75)	
**Ethnicity**									
Caucasian	285 (75.6)	312 (80)	0.1422	179 (75.21)	148 (72.91)	0.581	106 (76.26)	164 (87.7)	0.0068
Other	92 (24.4)	78 (20)		59 (24.79)	55 (27.09)		33 (23.74)	23 (12.3)	
Pediatric visit 3 days previous	90 (23.87)	61 (15.64)	0.0042	70 (29.41)	56 (27.59)	0.6723	20 (14.39)	5 (2.67)	< 0.0001
Sent by pediatrician	50 (13.26)	49 (12.56)	0.773	37 (15.55)	39 (19.21)	0.3097	13 (9.35)	10 (5.35)	0.1626
**Clinical characteristics**									
Sat O2									
In Room air	312 (93.98)	141 (98.6)	0.0097	205 (91.11)	107 (98.17)	0.0051	107 (100)	34 (100)	-
In oxygen therapy	16 (4.82)	2 (1.4)		16 (7.11)	2 (1.83)		0 (0)	0 (0)	
Unknown	4 (1.2)	0 (0)		4 (1.78)	0 (0)		0 (0)	0 (0)	
**Clinical tests**									
Chest x-ray	111 (29.92)	70 (17.99)	0.0001	94 (40)	54 (26.73)	0.0035	17 (12.5)	16 (8.56)	0.2479
Chest CT	2 (0.55)	1 (0.26)	0.3688	2 (0.86)	1 (0.52)	0.4063	0 (0)	0 (0)	-
Blood tests	218 (58.13)	135 (34.62)	< 0.0001	184 (77.97)	121 (59.61)	< 0.0001	34 (24.46)	14 (7.49)	< 0.0001
**Comorbidities**									
Prematurity	31 (8.22)	47 (12.05)	0.0795	26 (10.92)	29 (14.29)	0.2869	5 (3.6)	18 (9.63)	0.0355
Neurological	16 (4.24)	6 (1.54)	0.0248	13 (5.46)	5 (2.46)	0.1126	3 (2.16)	1 (0.53)	0.1773
Onco-haematological	9 (2.39)	8 (2.05)	0.752	8 (3.36)	6 (2.96)	0.8086	1 (0.72)	2 (1.07)	0.4225
Metabolic disease	4 (1.06)	0 (0)	0.0414	4 (1.68)	0 (0)	0.0838	0 (0)	0 (0)	-
Other comorbidity	48 (12.73)	35 (8.97)	0.094	35 (14.71)	25 (12.32)	0.4655	13 (9.35)	10 (5.35)	0.1626
No comorbidity	274 (72.68)	272 (69.74)	0.3695	159 (66.81)	127 (62.56)	0.352	115 (82.73)	145 (77.54)	0.2484

Table S2. Virus distribution stratified by center and setting

	Overall N (%) (N=767)				Inpatients N (%) (N=441)				Outpatients N (%) (N=326)			
	Padova	Bologna	P- value		Padova	Bologna	P- value		Padova	Bologna	P- value	
**SARS-CoV-2**	197 (52.25)	211 (54.10)	0.6081		83 (34.87)	32 (15.76)	< 0.0001		114 (82.01)	179 (95.72)	< 0.0001	
**RSV**	85 (22.55)	119 (30.51)	0.0126		83 (34.87)	119 (58.62)	< 0.0001		2 (1.44)	0 (0)	0.181	
**Adenovirus**	30 (7.96)	26 (6.67)	0.4921		22 (9.24)	23 (11.33)	0.4706		8 (5.76)	3 (1.6)	0.0323	
**Rhinovirus**	29 (7.69)	3 (0.77)	<0.0001	< 0.0001	26 (10.92)	2 (0.99)	< 0.0001	< 0.0001	3 (2.16)	1 (0.53)	0.1773	< 0.0001
**Influenza A e B**	20 (5.31)	24 (6.15)	0.6133		10 (4.2)	20 (9.85)	0.0188		10 (7.19)	4 (2.14)	0.026	
**Metapneumovirus**	10 (2.65)	7 (1.79)	0.4199		8 (3.36)	7 (3.45)	0.96		2 (1.44)	0 (0)	0.181	
**Coronavirus**	6 (1.59)	0 (0)	0.0138		6 (2.52)	0 (0)	0.024		0 (0)	0 (0)	-	
**Total**	377 (49.15)	390 (50.85)			238 (53.97)	203 (46.03)			139 (42.64)	187 (57.36)		

Table S3. Continuous clinical characteristics' distribution stratified by centre and setting

	**Overall, median (p25 - p75)**			**Inpatients, median (p25 - p75)**			**Outpatients, median (p25 - p75)**		
	**Padova**	**Bologna**	**P - value**	**Padova**	**Bologna**	**P - value**	**Padova**	**Bologna**	**P - value**
**Days of hospitalization**	4 (3 - 7)	5 (3 - 7)	0.5222	4 (3 - 7)	5 (3 - 7)	0.5222	-	-	-
**Temperature at home (°C)**	38 (36.8 - 38.7)	38 (37 - 38.8)	0.2815	37.95 (36.9 - 38.6)	38 (36.7 - 38.6)	0.9996	38 (36.7 - 38.7)	38.25 (37.5 – 39)	0.0677
**Systolic blood pressure (mmHg)**	103 (88 – 116)	102.5 (88 – 132.5)	0.7056	103 (87 – 115)	102.5 (88 – 132.5)	0.624	102 (98 - 120)	-	-
**Diastolic blood pressure (mmHg)**	65 (55 – 76)	61.5 (57.5 – 67)	0.6009	65 (51 – 77)	61.5 (57.5 - 67)	0.7107	68 (62 - 74)	-	-
**Heart rate (bpm)**	140 (126 - 155)	146.5 (133.5 - 160)	0.0075	145 (130 - 160)	150 (140 - 163.5)	0.022	130 (110 - 140)	132.5 (115 - 145)	0.3779
**Respiratory rate**	40 (30 - 60)	52 (45 - 60)	< 0.0001	40 (30 – 60)	55 (50 – 60)	< 0.0001	30 (25 – 40)	22 (20 – 36)	0.0194
**Sat O2 (%)**	99 (96 – 100)	98 (95 – 99)	< 0.0001	98 (95 – 100)	97 (94 – 98)	< 0.0001	99 (98 - 100)	99 (98 - 100)	0.6078
**Temperature in the ER (°C)**	37.5 (36.8 - 38.5)	37.85 (36.5 - 38.55)	0.7181	37.5 (36.8 - 38.5)	37.9 (36.5 - 38.65)	0.5435	-	-	-

Table S4 Clinical characteristics at admission to the PEDs stratified by centre, setting and type of virus

		**Age - mo.**			**Days of hospitalization**			**Temperature at home (°C)**			**Systolic blood pressure (mmHg)**			**Diastolic blood pressure (mmHg)**		
		**Padova**	**Bologna**	**P - value**	**Padova**	**Bologna**	**P - value**	**Padova**	**Bologna**	**P - value**	**Padova**	**Bologna**	**P - value**	**Padova**	**Bologna**	**P - value**
**Overall**	SARS-CoV-2	24.15 (6.74 - 100.92)	74.85 (13.58 - 129.18)	0.0002	4 (3 - 8)	4.5 (2 - 9)	0.9710	38 (36.7 - 38.5)	38.1 (37.5 - 39)	0.0031	107 (95 - 120)	102.5 (88 - 132.5)	0.9353	65 (55 - 74)	61.5 (57.5 - 67)	0.6264
	RSV	1.93 (1.18 - 5.48)	4.09 (2.26 - 10.89)	0.0002	5 (3 - 6)	5 (4 - 7)	0.4754	37.3 (36.8 - 38)	37.4 (36.6 - 38.5)	0.8947	90.5 (82 - 103)	-	-	57.5 (44 - 84)	-	-
	Adeno virus	19.89 (9.15 - 44.1)	24.2 (13.93 - 36.98)	0.5991	4 (3 - 5)	3 (2 - 5)	0.2598	39.5 (38.2 - 40)	39 (38 - 39.8)	0.6876	105 (96.5 - 109)	-	-	63.5 (53 - 67)	-	-
	Rhino-virus	5.84 (1.8 - 73.67)	42.90 (1.08 - 84.72)	0.9040	4 (3 - 7)	3 (2 - 4)	0.4144	38 (37 - 39)	37.5 (36 - 38.2)	0.2992	105 (67 - 108)	-	-	74 (66 - 96)	-	-
	Influenza A e B	23.08 (5.16 - 51.57)	13.21 (4.7 - 30.26)	0.4722	6 (3 - 9)	5 (3 - 7.5)	0.7733	39 (38.3 - 39.65)	38.2 (37.5 - 38.6)	0.0168	93 (62 - 110)	-	-	69.5 (63 - 97)	-	-
	Metapneumovirus	10.2 (2.56 - 24.69)	10.03 (6.07 - 25.38)	0.7327	4.5 (3.5 - 6)	4 (3 - 6)	0.5561	38.5 (38 - 38.7)	39 (38.2 - 40)	0.2281	101.5 (99 - 104)	-	-	57.5 (56 - 59)	-	-
	Corona virus	4.93 (1.9 - 17.41)	-	-	2.5 (2 - 3)	-	-	38.3 (37.2 - 38.5)	-	-	59 (59 - 59)	-		124 (124 - 124)	-	
**Inpatients**	SARS-CoV-2	18.75 (2.49 - 119.84)	35.84 (6.92 - 117.08)	0.3296	4 (3 - 8)	4.5 (2 - 9)	0.9710	37.8 (36.8 - 38)	38 (36.5 - 38.9)	0.3331	107.5 (93 - 120)	102.5 (88 - 132.5)	0.9487	64 (51 - 74)	61.5 (57.5 - 67)	0.8132
	RSV	1.87 (1.18 - 5.48)	4.09 (2.26 - 10.89)	< 0.0001	5 (3 - 6)	5 (4 - 7)	0.4754	37.3 (36.8 - 38)	37.4 (36.6 - 38.5)	0.9479	90.5 (82 - 103)	-	-	57.5 (44 - 84)	-	-
	Adeno virus	15.15 (9.15 - 35.74)	24.39 (15.61 - 38.95)	0.1526	4 (3 - 5)	3 (2 - 5)	0.2598	39.25 (38.2 - 40)	39 (38.5 - 39.8)	0.9773	102.5 (93 - 112)	-	-	63.5 (51 - 67)	-	-
	Rhino-virus	7.8 (1.38 - 79.74)	1.08 (1.08 - 1.08)	0.4039	4 (3 - 7)	3 (2 - 4)	0.4144	38.05 (37.2 - 39)	36.75 (36 - 37.5)	0.1282	87.5 (64.5 - 108)	-	-	74.5 (63 - 98)	-	-
	Influenza A e B	15.34 (0.95 - 30.16)	7.05 (2.93 - 29.8)	0.8776	6 (3 - 9)	5 (3 - 7.5)	0.7733	39.05 (39 - 39.8)	38.2 (37.5 - 38.5)	0.0076	85 (62 - 110)	-	-	76 (63 - 97)	-	-
	Metapneumovirus	7.74 (2.15 - 13.33)	10.03 (6.07 - 25.38)	0.3253	4.5 (3.5 - 6)	4 (3 - 6)	0.5561	38.3 (38 - 38.75)	39 (38.2 - 40)	0.2109	101.5 (99 - 104)	-	-	57.5 (56 - 59)	-	-
	Corona virus	4.93 (1.9 - 17.41)	-	-	2.5 (2 - 3)	-	-	38.3 (37.2 - 38.5)	-	-	59 (59 - 59)	-	-	124 (124 - 124)	-	-
**Outpatients**	SARS-CoV-2	25.97 (7.44 - 98.23)	81.62 (16.46 - 133.28)	0.0006	-	-	-	38 (36.6 - 38.6)	38.3 (37.5 - 39)	0.0145	100 (98 - 120)	-	-	68 (62 - 75)	-	-
	RSV	10.67 (5.28 - 16.07)	-	-	-	-	-	37.1 (36.2 - 38)	-	-	-	-	-	-	-	-
	Adeno virus	33.49	9.61 (0 - 28)	0.2616	-	-	-	39.5 (38 - 40)	38	0.3035	105.5 (105 - 106)	-	-	62.5 (55 - 70)	-	-

		(12.46 - 61.79)							(37.1 - 38.9)							
	Rhino-virus	3.41 (2.49 - 18.59)	84.72 (84.72 - 84.72)	0.3711	-	-	-	36.7 (36.3 - 39)	38.2 (38.2 - 38.2)	1.0000	108 (108 - 108)	-	-	74 (74 - 74)	-	-
	Influenza A e B	25.95 (16.33 - 65.97)	27.67 (21.13 - 53.56)	0.8320	-	-	-	38.75 (38.1 - 39.3)	38.35 (37.35 - 39.45)	0.5227	101 (101 - 101)	-	-	63 (63 - 63)	-	-
	Metapneumovirus	31.66 (24.69 - 38.62)	-	-	-	-	-	38.6 (38.6 - 38.6)	-	-	-	-	-	-	-	-
	Corona virus	-	-	-	-	-	-	-	-	-	-	-	-	-	-	-

		**Heart rate (bpm)**			**Respiratory rate**			**Sat O2 (%)**			**Temperature in the ER (°C)**		
		**Padova**	**Bologna**	**P - value**	**Padova**	**Bologna**	**P - value**	**Padova**	**Bologna**	**P - value**	**Padova**	**Bologna**	**P - value**
**Overall**	SARS-CoV-2	130 (107 - 140)	130 (111 - 140)	0.6155	30 (25 - 40)	22.5 (20 - 44)	0.2714	100 (99 - 100)	99 (98 - 100)	0.1677	37 (36.5 - 38)	36.55 (36.3 - 37.5)	0.2404
	RSV	149 (140 - 160)	150 (140 - 165)	0.5478	55 (40 - 60)	55 (50 - 60)	0.2597	96 (94 - 99)	96 (92.5 - 98)	0.2505	37.4 (36.8 - 38.4)	38 (36.5 - 38.65)	0.8313
	Adenovirus	135.5 (116.5 - 152)	126.5 (119 - 135)	0.4297	30 (25 - 40)	28 (24 - 40)	0.5136	97.5 (91 - 100)	98 (98 - 99)	0.6802	38.55 (38 - 39)	38.5 (37.9 - 39.5)	0.8161
	Rhino-virus	158.5 (136 - 168)	133 (132 - 134)	0.2279	40 (40 - 49)	52 (52 - 52)	0.2960	98 (94 - 100)	94.5 (92 - 97)	0.2685	37.75 (37.1 - 38.6)	-	-
	Influenza A e B	135 (126 - 150)	152.5 (136 - 160)	0.1250	25 (22 - 40)	62.5 (50 - 68)	0.0264	98 (97 - 100)	99 (98 - 100)	0.3148	38.25 (37 - 39.4)	37.95 (37.5 - 38.3)	0.6228
	Metapneumovirus	148.5 (135.5 - 170)	165 (150 - 180)	0.6004	42.5 (30 - 60)	60 (50 - 60)	0.4392	96 (92 - 98)	97 (94.5 - 97.5)	0.7210	37.75 (37.35 - 38.25)	38.4 (38.4 - 38.4)	0.4367
	Coronavirus	160 (158 - 170)	-	-	50 (45 - 60)	-	-	98 (93 - 100)	-	-	38.55 (37.2 - 39.3)	-	-
**Inpatients**	SARS-CoV-2	130 (105 - 146)	145 (130 - 160)	0.1264	30 (25 - 38)	65 (20 - 70)	0.3918	99 (99 - 100)	100 (99 - 100)	0.6135	37 (36.5 - 38)	36.6 (36.3 - 37.8)	0.4037
	RSV	149 (140 - 160)	150 (140 - 165)	0.5938	55 (40 - 60)	55 (50 - 60)	0.3034	96 (94 - 99)	96 (92.5 - 98)	0.1799	37.4 (36.8 - 38.4)	38 (36.5 - 38.65)	0.8313
	Adenovirus	136 (130 - 160)	122.5 (115 - 130)	0.2851	30 (25 - 40)	32 (24 - 40)	0.5772	97 (90 - 99.5)	98 (98 - 99)	0.5034	38.55 (38 - 39)	38.5 (37.9 - 39.5)	0.8161
	Rhino-virus	158.5 (137.5 - 168)	134 (134 - 134)	0.3666	40 (40 - 50)	52 (52 - 52)	0.3404	98 (94 - 100)	92 (92 - 92)	0.3050	37.75 (37.1 - 38.6)	-	-
	Influenza A e B	135 (129 - 158)	138 (130 - 170)	0.8594	32.5 (22 - 40)	65 (60 - 68)	0.0265	99 (96 - 100)	98 (97 - 99)	0.6468	38.25 (37 - 39.4)	37.7 (37.5 - 38.2)	0.5951
	Metapneumovirus	155.5 (139 - 180)	165 (150 - 180)	1.0000	52.5 (35 - 62.5)	60 (50 - 60)	0.7540	96.5 (92.5 - 98.5)	97 (94.5 - 97.5)	1.0000	37.75 (37.35 - 38.25)	38.4 (38.4 - 38.4)	0.4367
	Coronavirus	160 (158 - 170)	-	-	50 (45 - 60)	-	-	98 (93 - 100)	-	-	38.55 (37.2 - 39.3)	-	-
**Outpatients**	SARS-CoV-2	130 (110 - 138)	125 (110 - 140)	0.8135	32.5 (29 - 41)	22 (20 - 40)	0.0740	100 (99 - 100)	99 (98 - 100)	0.0459	-	-	-
	RSV	142.5 (132 - 153)	-	-	47.5 (40 - 55)	-	-	90 (85 - 95)	-	-	-	-	-
	Adenovirus	135 (100 - 148)	131.5 (123 - 140)	1.0000	25 (25 - 40)	28 (28 - 28)	1.0000	99 (96 - 100)	98.5 (98 - 99)	0.8635	-	-	-

	Rhino-virus	142.5 (119 - 166)	132 (132 - 132)	1.0000	40 (40 - 48)	-	-	99 (98 - 100)	97 (97 - 97)	0.3711	-	-	-
	Influenza A e B	135 (119 - 148)	157.5 (152.5 - 160)	0.0170	25 (25 - 30)	22 (22 - 22)	0.2749	98 (97 - 98)	100 (100 - 100)	0.0128	-	-	-
	Metapneumovirus	128 (110 - 146)	-	-	35 (30 - 40)	-	-	94 (92 - 96)	-	-	-	-	-
	Coronavirus	-	-	-	-	-	-	-	-	-	-	-	-

Table S5. Use of resources distribution.

	Overall N (%)			Inpatients N (%)			Outpatients N (%)		
	**Padova**	**Bologna**	**P- value**	**Padova**	**Bologna**	**P- value**	**Padova**	**Bologna**	**P- value**
	**(N=377)**	**(N=390)**		**(N=238)**	**(N=203)**		**(N=139)**	**(N=187)**	
**Needing for O2 therapy**									
No	272 (59.78)	308 (76.62)	-	137 (43.35)	127 (57.47)	< 0.0001	135 (97.12)	181 (100)	0.0347
O2 tp low flow	84 (18.46)	66 (16.42)		81 (25.63)	66 (29.86)		3 (2.16)	0 (0)	
O2 tp high flow	65 (14.29)	24 (5.97)		65 (20.57)	24 (10.86)		0 (0)	0 (0)	
O2 tp Venturi mask (reservoir)	22 (4.84)	0 (0)		21 (6.65)	(0)		1 (0.72)	0 (0)	
Mechanical ventilation	3 (0.66)	1 (0.25)		3 (0.95)	1 (0.45)		0 (0)	0 (0)	
NIV	9 (1.98)	3 (0.75)		9 (2.85)	3 (1.36)		0 (0)	0 (0)	
**Antiviral**									
No	375 (99.73)	372 (100)	0.5027	236 (99.58)	187 (100)	0.559	139 (100)	185 (100)	-
Yes	1 (0.27)	0 (0)		1 (0.42)	0 (0)		0 (0)	0 (0)	
**Antibiotics**									
No	282 (74.8)	337 (90.11)	< 0.0001	165 (69.33)	173 (92.02)	< 0.0001	117 (84.17)	164 (88.17)	0.2971
Yes	95 (25.2)	37 (9.89)		73 (30.67)	15 (7.98)		22 (15.83)	22 (11.83)	
**Corticosteroids**									
No	319 (85.29)	356 (94.68)	< 0.0001	194 (82.2)	177 (93.65)	0.0004	125 (90.58)	179 (95.72)	0.0623
Yes	55 (14.71)	20 (5.32)		42 (17.8)	12 (6.35)		13 (9.42)	8 (4.28)	
**Antimycotics**									
No	373 (99.73)	375 (99.73)	0.5007	235 (99.58)	190 (100)	0.554	138 (100)	185 (99.46)	0.5741
Yes	1 (0.27)	1 (0.27)		1 (0.42)	0 (0)		0 (0)	1 (0.54)	
**Paracetamol**									
No	240 (63.83)	236 (87.73)	< 0.0001	144 (60.76)	166 (94.86)	< 0.0001	96 (69.06)	70 (74.47)	0.3713
Yes	136 (36.17)	33 (12.27)		93 (39.24)	9 (5.14)		43 (30.94)	24 (25.53)	
**FANS**									
No	339 (90.16)	317 (96.65)	0.0007	218 (91.98)	182 (100)	< 0.0001	121 (87.05)	135 (92.47)	0.1307
Yes	37 (9.84)	11 (3.35)		19 (8.02)	0 (0)		18 (12.95)	11 (7.53)	
**Chloroquine**									
Chloroquine	2 80.53)	0 (0)	0.0628	1 (0.42)	0 (0)	0.2574	1 (0.72)	0 (0)	0.1409
Hydroxychloroquine (Plaquenil)	1 (0.27)	3 (0.80)		1 (0.42)	1 (0.53)		0 (0)	2 (1.08)	
No	372 (99.20)	372 (99.20)		235 (99.16)	188 (99.47)		137 (99.28)	184 (98.92)	
**Other drug**									
No	304 (81.07)	323 (87.06)	0.0253	185 (78.39)	151 (81.18)	0.4795	119 (85.61)	172 (92.97)	0.0301
Yes	71 (18.93)	48 (12.94)		51 (21.61)	35 (18.82)		20 (14.39)	13 (7.03)	
**Days in ICU**									
0	229 (95.42)	202 (96.19)	0.6838	226 (95.36)	195 (96.06)	0.7186	-	-	-
At least 1	11 (4.58)	8 (3.81)		11 (4.64)	8 (3.94)		-	-	
